# Phylogenomics: Gene Duplication, Unrecognized Paralogy and Outgroup Choice

**DOI:** 10.1371/journal.pone.0004568

**Published:** 2009-02-23

**Authors:** Scott William Roy

**Affiliations:** National Center for Biotechnology Information, National Library of Medicine, National Institutes of Health, Bethesda, Maryland, United States of America; University of Hyderabad, India

## Abstract

Comparative genomics has revealed the ubiquity of gene and genome duplication and subsequent gene loss. In the case of gene duplication and subsequent loss, gene trees can differ from species trees, thus frequent gene duplication poses a challenge for reconstruction of species relationships. Here I address the case of multi-gene sets of putative orthologs that include some unrecognized paralogs due to ancestral gene duplication, and ask how outgroups should best be chosen to reduce the degree of non-species tree (NST) signal. Consideration of expected internal branch lengths supports several conclusions: (i) when a single outgroup is used, the degree of NST signal arising from gene duplication is either independent of outgroup choice, or is minimized by use of a maximally closely related post-duplication (MCRPD) outgroup; (ii) when two outgroups are used, NST signal is minimized by using one MCRPD outgroup, while the position of the second outgroup is of lesser importance; and (iii) when two outgroups are used, the ability to detect gene trees that are inconsistent with known aspects of the species tree is maximized by use of one MCRPD, and is either independent of the position of the second outgroup, or is maximized for a more distantly related second outgroup. Overall, these results generalize the utility of closely-related outgroups for phylogenetic analysis.

## Introduction

Accurate phylogenetic inference is thwarted by the presence of conflicting signals in the data (e.g., [1]–[3]), a problem that has received a large amount of theoretical and experimental attention over the past few years (e.g., [4]–[7]). One important general source of problems in phylogenetic analysis is gene duplication (e.g., [8]–[10]).

In particular, loss of different members of an ancestral duplicate pair in different species can lead to a gene tree that does not reflect the species tree [11]–[13]. Recent studies in yeast and teleost fish [14]–[16] suggest that such reciprocal loss of gene duplicates following genome duplication may be a common phenomenon, raising the specter of significant conflicts between gene trees and species trees in such lineages. Such cases are particularly troublesome since they are expected to pass the most common bioinformatics test for one-to-one orthologs: genes from different species that are each other's best reciprocal BLAST hit. While under such circumstances every attempt to identify and eliminate such paralogs from analyses should be made, it remains likely that some paralogs will persist in many-locus sets of putative orthologs. Given the importance of gene duplication in the evolution of eukaryotic genomes in general and of several lineages of great interest in particular [17]–[19], understanding such challenges is useful for correct reconstruction of evolutionary history.

Recently, Rogozin et al. [20] suggested that in the case of whole-genome duplication, use of a closely related outgroup – specifically one that diverged from the species of introns following the duplication – might lead to incorrect species tree reconstruction. Thus, in stark contrast to the general case in the absence of gene duplication, use of a more distant outgroup might be more likely to yield an accurate tree. A great deal of theoretical and empirical work has vindicated taxonomic sampling to reduce branch lengths, including use of closely related outgroups [21]–[23]; however the case of multi-locus analyses in the presence of differential loss of ancestral gene duplicates has not been addressed to my knowledge. Here I address the issue of outgroup choice in the presence of NST signal arising from subsets of unrecognized paralogs genes within many-gene sets of putative orthologs.

## Analysis and Discussion

### Phylogenetic reconstruction in the presence of unrecognized paralogs

I consider the problem of resolving the relationship between three species/groups by studying many-gene sets of putative one-to-one orthologs, in which some genes have been duplicated in the ancestor of the three species/groups of interest. Following such an ancestral duplication, there are three possible outcomes. Some studied species might retain both duplicates, in which case the gene will not be a one-to-one ortholog, and thus should be recognized and discarded. If not, the two most closely related species/groups might retain the same duplicate copy (either by independent loss of the other duplicate, or loss of one duplicate in an ancestor of the two species/groups), in which case the topologies of the gene and species trees will be identical, and thus the gene duplication is not expected to pose problems to reconstruction of the species tree. Finally, all three species/groups might lose one duplicate copy, but the two most closely related groups lose different (reciprocal) copies, in which case the species and gene trees will have different topologies. It is that third case that I address here.

The problem is treated under the following conditions and assumptions. First, I consider phylogenetic analysis of concatenated alignments across putative orthologs. Notably, the present arguments do not apply for methods such as matrix representation parsimony, in which trees are reconstructed from individual gene alignments. In such cases the length of the branch supporting the incorrect (non-species) grouping is not of consequence, but only the topology itself. Second, I assume that in general shorter NST internal branches are preferable to long ones, since such branches are expected to generally experience fewer changes and thus contribute less NST signal.

### Gene and species trees under gene duplication and loss

**Figure 1:**
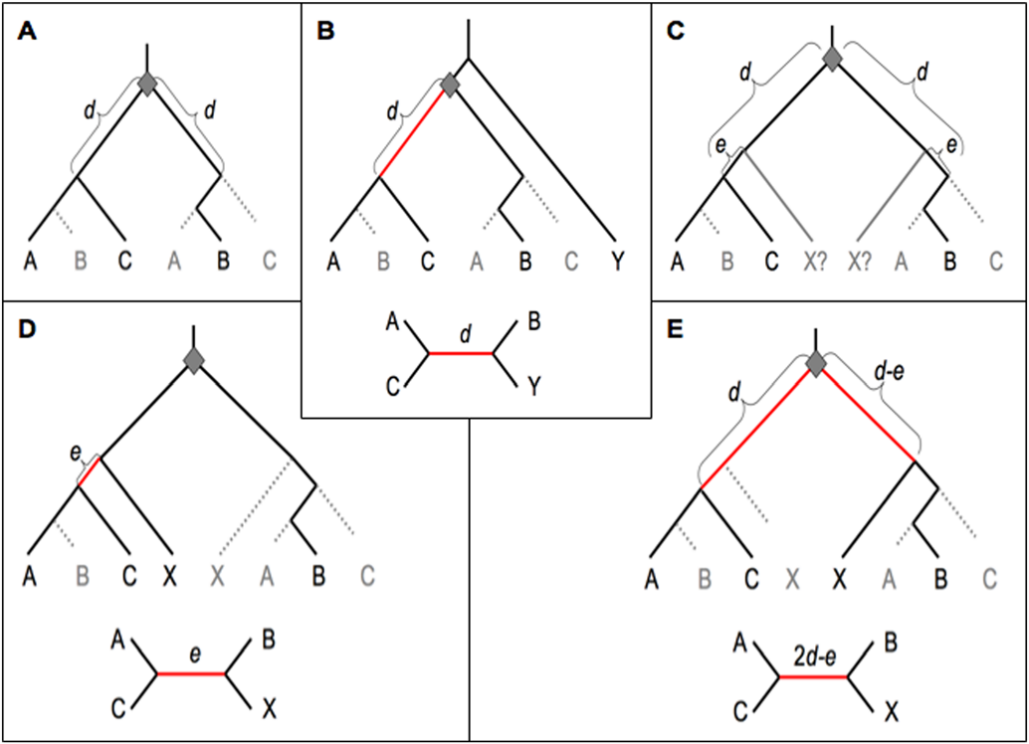
Differential loss of ancestral duplicates and gene trees with single outgroups, for different phylogenetic positions of the outgroup. a) The general case in which duplication (grey diamond) followed by differential gene loss (dotted grey lines) leads to a closer relationship between gene copies from species A and C, in contrast to the species relationship. b) The case for a single pre-duplication outgroup Y. c) The general case for a post-duplication outgroup X diverged time *e* before the C/AB split. d) X and C retain the same duplicate copy. e) X and B retain the same duplicate copy. Both rooted and unrooted trees are shown. Red branches indicate the NST internal branch, supporting an A+C group.

Gene duplication and differential subsequent gene loss can lead to gene trees that do not reflect the species tree. The greater the strength of this alternative (NST) signal, the greater the chance of recovering the wrong tree in multi-locus analyses. The general case is illustrated in Figure 1a. Species C represents an outgroup to species A and B. A gene duplication (grey diamond) occurred at a time *d* before the C/AB split. Both duplicates were retained all the way to the A/B split. The three species then each lost one of the two duplicates (dotted grey lines), returning to a single-copy state (solid black lines). Although species C represents an outgroup to species A and B, retention of the same duplicate copy in species A and C (left side) but the other copy in species B (right side) leads to a case in which the remaining copies in A and C are most closely related. (Note that, throughout, all figures consider the case in which C and A retain the same duplicate. For each case there is an equivalent case in which C and B (but not A) retain the same duplicate).

### One outgroup

I next consider gene trees with a single outgroup. Such an outgroup may diverge from the ABC ancestor before the duplication (Figure 1b) or may diverge after the duplication and subsequently lose one duplicate copy (Figure 1c–e; if the outgroup retains both duplicates, the gene will be recognized as not a one-to-one ortholog, and should be discarded). Figure 1c gives the general scenario, and Figure 1d and e illustrate the cases in which either duplicate is lost in species X, with both rooted and simplified unrooted trees shown.

**Figure 2:**
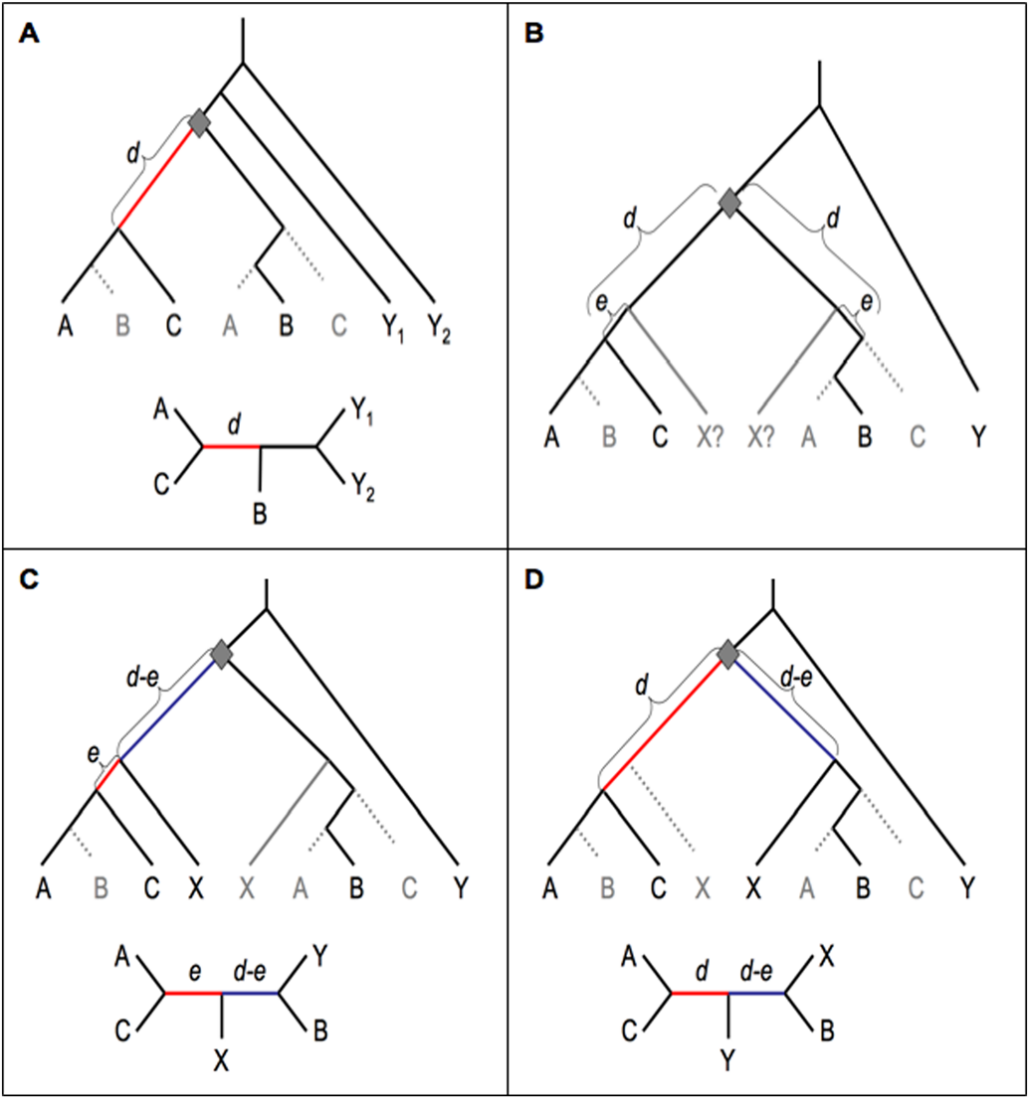
Gene trees with two outgroups, including at least one pre-duplication outgroup. a) Two pre-duplication outgroups Y_1_ and Y_2_. b) The general case for one pre-duplication outgroup Y and one post-duplication outgroup X. c) X and C retain the same duplicate copy. d) X and B retain the same duplicate copy. Red branches indicate the NST internal branch supporting an A+C clade. Blue branches indicate the known non-species tree (KNST) branch, contradicting the known A+B+C group.

**Figure 3:**
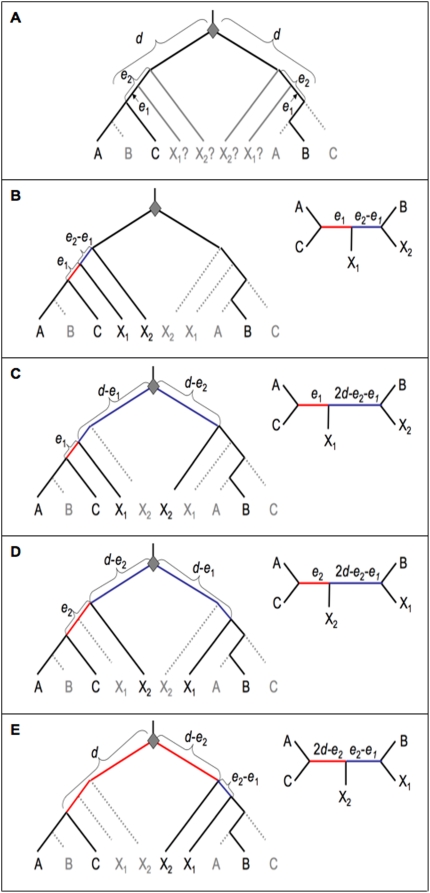
Gene trees with two post-duplication outgroups. a) The general case for two post-duplication outgroups X_1_ and X_2_, diverged *e*_1_ and *e*_2_ before that C/AB split, respectively. b) X_1_, X_2_ and C retain the same duplicate copy. c) X_1_ and C (but not X_2_) retain the same duplicate copy. d) X_2_ and C (but not X_1_) retain the same duplicate copy. e) X_1_, X_2_ and B retain the same duplicate copy. Red and blue indicate NST and KNST branches, respectively.

As Figure 1b,d,e shows, the four-taxa gene tree consistently contradicts the species tree regardless of the position of the outgroup relative to the duplication event (pre- or post-) and regardless of which duplicate is lost in a post-duplication outgroup. That is, in each case the gene tree contains an A+C group (red branches), in contrast to the true species grouping A+B. How does outgroup position affect the amount of NST signal? NST signal is due to changes occurring along the aberrant internal branch (red in Figures 1, 2, 3). The shorter this internal branch, the fewer expected genetic changes and the less NST signal, so in general outgroups that minimize NST internal branch length are preferable.

Using a pre-duplication outgroup (Figure 1b), the length of the NST internal branch is *d*. Using a post-duplication outgroup the internal branch length is either *e*, the time between the X/ABC and C/AB divergences (if X and C retain the same duplicate copy; Figure 1d) or 2*d-e* (if X and C retain different copies; Figure 1e). If these two possibilities are equally likely, the average branch length is *d*, equal to the value for a pre-duplication outgroup.

What if one copy is more likely to be retained? In this case, it is more likely that X and C retain the same duplicate, and the expected NST internal branch length will be less than *d*. In the case where the two duplicates' probabilities of retention differ by a factor *r*, the two duplicates have probabilities 0.5(1±*r*) along each branch, and, assuming independent loss along each branch, the probability that X and C retain the same duplicate is [0.5(1+*r*)]^2^+[0.5(1−*r*)]^2^ = 0.5(1+*r* ^2^), giving an average internal branch length of *d*-*r^2^*(*d-e*), which is less than *d*. Thus in this case: (i) a post-duplication outgroup yields shorter average internal branches, and (ii) later-diverging post-duplication outgroups yield shorter internal branches than do more distant ones (i.e. small values of *e*).

In total then, in the case of a single outgroup, a post-duplication outgroup is a better choice for several reasons: (i) the outgroup may retain both duplicates, allowing detection of the otherwise cryptic ancestral gene duplication; (ii) the well-known general advantages of using closely related outgroups; and (iii) lesser or equal NST signal issuing from cases of differential loss of ancestral duplicates.

### Two outgroups

How do these considerations change when two outgroups are used? In this case, zero, one, or two outgroups may predate the duplication event (Figure 2,3). If both outgroups predate the duplication event, the average NST internal branch length is again *d* (Figure 2a). With one pre- and one post-duplication outgroup (Figure 2b), the internal branch length is either *e* (if X and C retain the same copy; Figure 2c) or *d* (if X and C retain different copies; Figure 2d). Thus in this case the average internal branch length is *e* X 0.5(1+*r^2^*)+*d* X 0.5(1−*r^2^*) = 0.5((*d+e*)−*r* ^2^(*d−e*)). This is less than or equal to *d* (since *e*≤*d*), thus (i) mixed pre/post duplication outgroups are expected to lead to less NST signal than are two pre-duplication outgroups, and (ii) a more closely related post-duplication outgroup is expected to lead to less NST signal than for a more distant one (i.e., small *e* value).

The case is somewhat more complicated for two post-duplication outgroups. Figure 3a illustrates the general case, with outgroups X_1_ and X_2_ that diverged at times *e*_1_ and *e*_2_ before the C/AB divergence, respectively (with *e*_1_<*e*_2_<*d*). Now there are four possibilities based on which duplicate copies are retained by X_1_ and X_2_ relative to C. If X_1_ and C retain the same copy (with probability 0.5(1+*r^2^*)), the internal NST branch is *e*_1_ (regardless of which duplicate is retained in X_2_; Figure 3b,c). If X_1_ and C retain different copies (with probability 0.5(1−*r* ^2^)), the issue becomes more complicated still. In this case, the NST internal branch is *e*_2_ if X_2_ and C retain the same copy (Figure 3d), or 2*d*-*e_2_* if X_2_ and C retain different copies (Figure 3e).

Which of these two scenarios (i.e. Figure 3d and e) is more likely? The probability of X_2_ retaining the same copy as C is equal to the probability that X_2_ and C (but not X_1_) retain the most likely duplicate, 0.5(1+*r*) X 0.5(1+*r*) X 0.5(1−*r*), plus the probability that X_2_ and C (but not X_1_) retain the less likely duplicate, 0.5(1−*r*) X 0.5(1−*r*) X 0.5(1+*r*). This sum is simply 0.25(1−*r* ^2^). The probability of X_2_ and X_1_ (but not C) retaining the same copy is identical, following the same reasoning. Thus the scenarios in Figure 3d and 3e have equally probability. The total expected NST internal branch length is then equal to (i) the probability that X_1_ and C retain the same duplicate, 0.5(1+*r* ^2^), times *e*_1_, plus (ii) the probability that X_1_ and C retain different duplicates, 0.5(1−*r* ^2^), times the average of *e*_2_ and 2*d*-*e*_2_ (which is *d*). This yields a total average expected NST internal branch length of 0.5((*d+e*_1_)−*r* ^2^(*d−e*_1_)). This value is equal to the expectation for one post-duplication outgroup diverging at time *e*_1_ and one pre-duplication outgroup (as shown above). Thus, using multiple outgroups, NST signal is minimized by using one closely-related post-duplication outgroup, while the position of the second outgroup (whether pre-duplication, or diverging any time between the duplication and C/AB divergence) does not affect the degree of NST signal in this model.

### Maximizing signal for gene tree branches contradicting known species relationships

In the case of multiple outgroups, a second consideration comes into play. If the additional species (Y, X_1_, X_2_) are confidently known to be true outgroups to an A+B+C clade, then the possibility exists for detecting gene trees that contradict known species relationship, allowing for exclusion of such suspect genes from multi-locus analyses. For instance, for the mixed pre/post duplication outgroup case shown in Figure 2c, the gene tree contains a known non-species tree (KNST) branch (shown in blue) dividing A+C+X from B+Y, which contradicts the known A+B+C group. Since this contradictory signal is useful in detecting suspicious gene trees, optimal outgroup combinations will maximize, not minimize, the length of the KNST.

Firstly, and most clearly, the presence/absence of a KNST branch differs between different outgroup combinations. For two pre-duplication outgroups, the gene tree still contains an A+B+C group (Figure 2a), thus there is no KNST branch. By contrast, all cases with at least one post-duplication outgroup (Figure 2c,d and Figure 3b–e, excepting post-duplication pairs for which *e*_1_ = *e*_2_) contain a KNST branch, thus outgroup combinations that include at least one post-duplication outgroup are preferred.

For mixed pre/post duplication outgroup pairs the length of the KNST branch is *d-e*_1_ (Figure 2c,d) regardless of which duplicate is retained in species X. This value is maximized for small *e*_1_, again supporting usage of a closely related outgroup. For two post-duplication outgroups, the KNST branch length is *e*_2_-*e*_1_ (if both outgroups retain the same duplicate; Figure 3b,e) or 2*d-e*_2_ *-e*_1_ (if different duplicates are retained; Figure 3c,d). Thus if both duplicates are equally likely to be retained, the average KNST branch length is *d-e*_1_, the same as for mixed pre/post duplication outgroups. If instead duplicates are not equally likely to be retained, the expected average length is smaller than for mixed outgroups: 0.5(1+*r* ^2^)(*e*_2_-*e*_1_)+0.5(1−*r* ^2^)(2d-*e*_2_ *-e*_1_) = *d-e*_1_ - *r* ^2^(*d-e*_2_). In this case, KNST branch length is maximized when one outgroup divergence is maximally close (i.e. coincident with the C/AB divergence: *e*_1_ = 0) and the other outgroup is coincident with the duplication (*d* = *e*_2_). Thus, under unequal probabilities of retention, KNST branch length is maximized for one very close outgroup and one more distant one. As such, depending on the relative probabilities of retention of the two duplicates, the ability to detect aberrant internal branches may provide an exception to the generally better expected performance of maximally closely-related outgroups for phylogenetic analysis.

### Caveats to the study

Two limitations of this study are worthy of note. First, only the relationship between three species is considered; however, as more and more recent divergences become resolved, many phylogenetic problems of central interest increasingly reduce to resolution of the relationship between three or a small number of deeply divergent groups. Second, I only consider the case with one or two outgroups; while this is an unrealistically small number of outgroups for single-gene studies, for genome-wide studies such considerations become more important, particularly given the reduction in numbers of detectable orthologs as more taxa are added.

### Concluding remarks

This discussion of outgroup choice in the presence of differentially retained ancestral gene duplicates yields two arguments supporting the use of closely-related outgroups: (i) for single outgroups, a maximally closely-related post-duplication outgroup either as good as or better than more distant outgroups at reducing non-species tree signal; and (ii) for two outgroups are used, non-species tree signal is minimized when one outgroup is maximally closely-related to the in groups, while the second outgroup position matters less. On the other hand, while the position of the second outgroup position is less important, in the case of different probabilities of retention among duplicates, power to detect gene trees that conflict with known species relationships is maximized when the second outgroup is coincident with or before the duplication event. These findings extend the utility of closely-related outgroups to reducing conflicting signals arising from gene duplication under certain conditions.
